# A case of mesenteric venous thrombosis complicating appendiceal diverticulitis

**DOI:** 10.1186/s40792-023-01592-0

**Published:** 2023-01-26

**Authors:** Jun Ichikawa, Kosuke Toda, Haruku Fujita, Kenjiro Hirai, Hidenori Ohe, Hidekazu Yamamoto

**Affiliations:** 1 Department of Surgery, Shiga General Hospital, 5-4-30 Moriyama, Moriyamashi, Shiga 524-8524 Moriyama City, Japan; 2 Department of Surgery, Graduate School of Medicine, Kyoto University, 53 Kawara-Cho Shogoin, Sakyo-Ku, Kyoto, 606-8507 Japan; 3 Department of Surgery, Japanese Red Cross Otsu Hospital, 1-35 Nagara, Otsu City, Shiga 520-8511 Japan

**Keywords:** Mesenteric venous thrombosis, Acute appendicitis, Appendiceal diverticulitis, Laparoscopic appendectomy, Specimen

## Abstract

**Background:**

Mesenteric venous thrombosis (MVT) and appendiceal diverticulitis are rare diseases. There has been no previous report on MVT complicating appendiceal diverticulitis. Herein, we report the first case of MVT complicating appendiceal diverticulitis.

**Case presentation:**

A 70-year-old male patient with right lower abdominal pain presented to our hospital. Abdominal contrast-enhanced computed tomography (CT) suggested MVT complicating appendiceal diverticulitis. Initially, we started conservative treatment with antibacterial drugs, but on the 2nd hospital day his general condition deteriorated due to sepsis that seemed to be caused by appendiceal diverticulitis. Therefore, we performed laparoscopic appendectomy. Histopathological findings of the specimen showed appendiceal diverticulitis. After the operation, he gradually improved. He was discharged on the 30th hospital day.

**Conclusions:**

We report a successfully treated case of MVT complicating appendiceal diverticulitis by surgical intervention. This is the first case of MVT complicating appendiceal diverticulitis.

## Background

Mesenteric venous thrombosis (MVT) is a rare and fatal condition that leads to intestinal congestion and ultimately to bowel necrosis. Appendiceal diverticulitis is also a rare disease. Appendiceal diverticulitis has a higher possibility of complications, such as perforation and abscess formation, than normal acute appendicitis. Recently, there have been some reports about MVT complicating acute appendicitis. However, MVT complicating appendiceal diverticulitis is quite rare. We experienced a case of MVT complicating appendiceal diverticulitis that we treated by surgical intervention. This is the first case of MVT complicating appendiceal diverticulitis.

## Case presentation

**Fig. 1:**
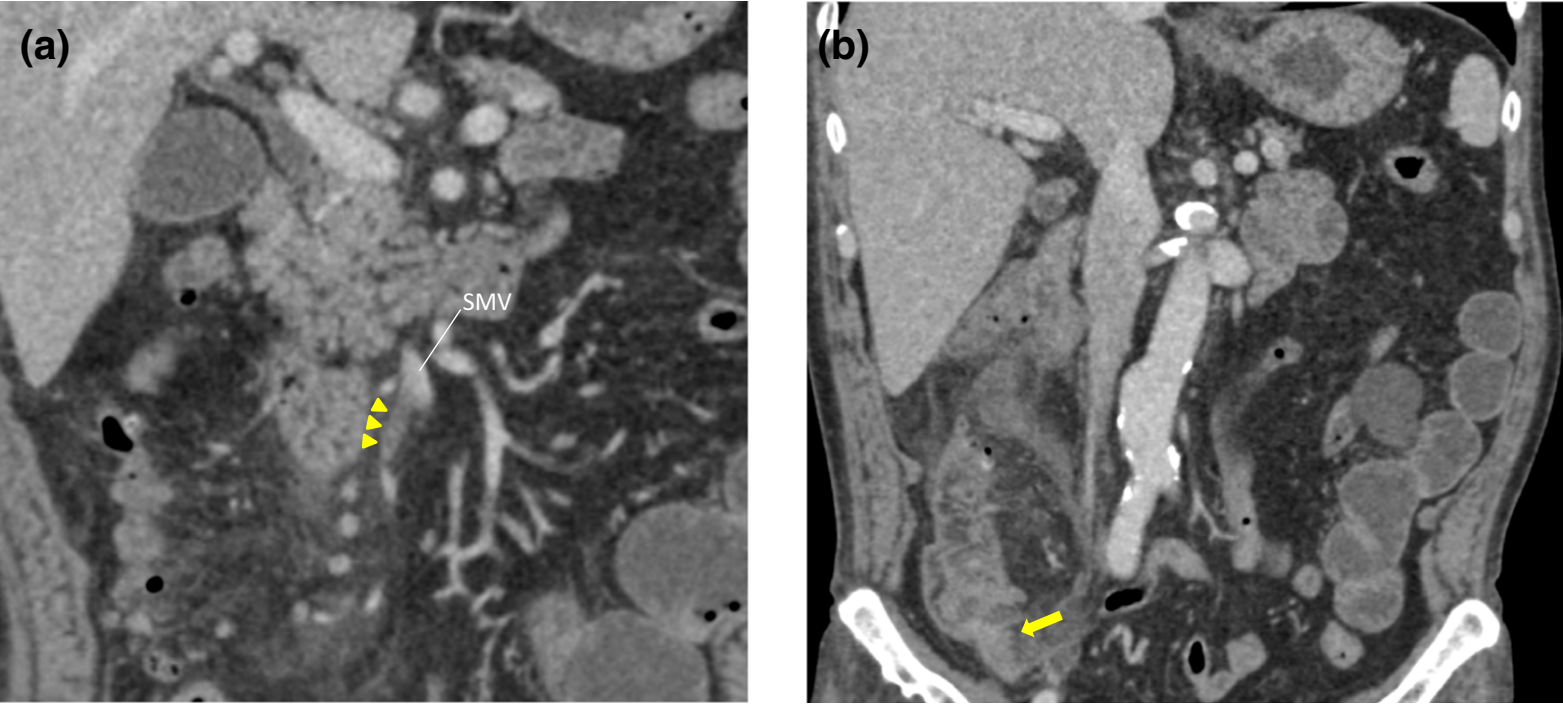
**a** Abdominal contrast-enhanced CT on admission. Thrombus is found from the ileocolic vein to the superior mesenteric vein. Arrowheads: mesenteric vein thrombus. **b** Abdominal contrast-enhanced CT on admission. Periappendiceal fat standings and unevenness of the appendix wall are found. Appendiceal diverticulitis is suspected. Arrow: unevenness of the appendix wall

**Fig. 2:**
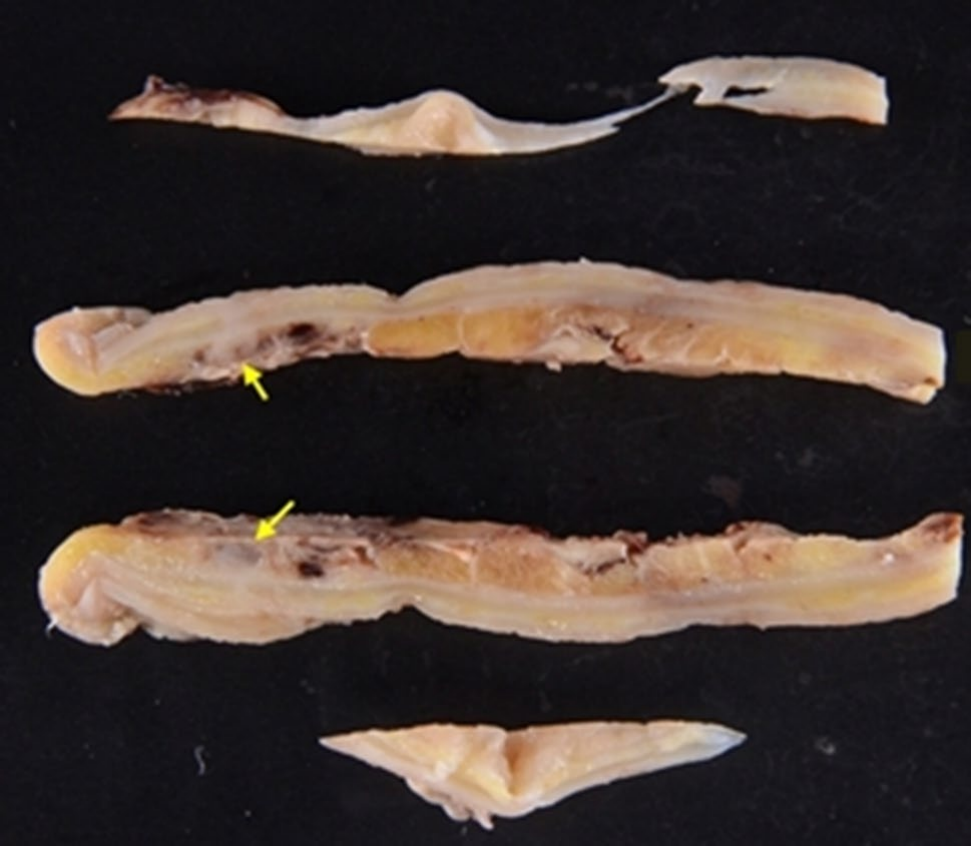
The excised specimen. The appendix of the resected specimen has diverticula. Arrows: appendix diverticulum

A 70-year-old man presented to our hospital with right lower abdominal pain and malaise. His past history was significant for coronary artery disease status postmyocardial infarction. On admission his temperature was 37.1 °C, pulse was 107 beats per minute, blood pressure was 132/77 mmHg, and oxygen saturation level was 95% room air. He exhibited right lower quadrant tenderness without peritoneal irritation. In laboratory data, the white blood cell count (WBC) was 10,000/μL, and the platelet count (PLT) was 36,000/μL. Alkaline phosphatase (ALP) was 554 IU/L, gamma-glutamyl transpeptidase (GGT) was 147 IU/L, total bilirubin (T-Bil) was 7.1 mg/dL, and C-reactive protein (CRP) was 26.35 mg/dL. High inflammatory responses, elevated liver enzyme, and hyperbilirubinemia were observed. Prothrombin time of international normalized ration (PT-INR) was 1.25, activated partial thromboplastin time (APTT) was 42 s. No significant abnormalities in the blood coagulation tests were observed. Abdominal contrast-enhanced computed tomography (CT) showed a thrombus from the ileocolic vein to the superior mesenteric vein. No ischemic change was found in the intestinal tract. Periappendiceal fat standings and unevenness of the appendix wall were suggestive of appendiceal diverticulitis (Fig. 1a, b). Neither appendicolith nor neoplasma were found. We suspected MVT complicating appendiceal diverticulitis. We initially started conservative treatment with antibiotics. However, on the 2nd hospital day his general condition deteriorated due to sepsis that seemed to be caused by appendiceal diverticulitis. His temperature was 37.3 °C, pulse was 93 beats per minute, respirations were 30 per minute, blood pressure was 101/61 mmHg, and oxygen saturation level was 94% under 6 L/min via mask. In laboratory date, WBC was 9800/μL, PLT was 4400/μL, ALP was 402 IU/L, GGT was 98 IU/L, T-Bil was 7.2 mg/dL, CRP was 25.86 mg/dL, PT-INR was 1.32, and APTT was 46 s. As no significant laboratory data changes but deterioration was recognized, conservative treatment was considered ineffective. We immediately performed laparoscopic appendectomy**.** Laparoscopy revealed swollen and reddish appendix with diverticulum without perforation. No ischemic changes were found in the small and large intestinal tract. We resected the appendix without complications. Histopathological findings of the specimen showed appendiceal diverticulitis (Fig. 2). He developed a postoperative abscess, which improved with antibiotics, and was physically inactive. He was hospitalized for rehabilitation. On the 27th hospital day follow-up contrast-enhanced CT showed resolution of MVT. Finally, he was discharged on the 30th hospital day.

## Discussion

MVT is rare; it is diagnosed in 1 per approximately 1500–5000 inpatients and 1 per 1000 emergently admitted patients [1]. The etiology of developing MVT includes genetic factors (such as antithrombin III deficiency and protein C/S deficiency), malignant tumors, inflammatory abdominal diseases, hypercoagulability (such as abdominal surgery), and other idiopathic conditions. Diverticulitis and appendicitis are the most common inflammatory abdominal diseases that cause MVT [1]. MVT may influence venous perfusion with disease progression and leads to bowel congestion, finally causing intestinal necrosis and leading to a fatal outcome. Recently, it was reported that the disease progression of MVT is slower than that of portal thrombosis, and that the risk of mortality is lower [2]. However, as MVT accounts for 6–9% of the causes of acute intestinal ischemia, early diagnosis and treatment are necessary [1].

Concerning diagnosis, there are no MVT-specific symptoms or hematological data. Therefore, it is difficult to make a diagnosis based on these findings alone. Some studies reported that hematology on MVT showed an increase in the liver enzyme level or hyperbilirubinemia [3, 4]. In fact, similar findings were observed in this case as well. Furthermore, contrast-enhanced CT is useful for diagnosis. The accuracy of CT in MVT diagnosis is reportedly 90% [5].

Treatment for MVT consists of two methods: primary disease treatment and anticoagulant therapy. In the case of appendicitis-related MVT, many previous studies recommended early surgical treatment for appendicitis from the viewpoint of early removal of an etiological factor [3, 4]. On the other hand, responders to conservative treatment were also reported. Furthermore, some studies recommended anticoagulant therapy for MVT from the viewpoint of thrombus extension-related intestinal necrosis prevention [5, 6]. However, further studies about anticoagulant therapy for MVT are required.

Diverticulum of the appendix is also rare. It is reportedly observed in 0.004 to 2.1% of patients who underwent appendectomy [7]. This disorder is classified into congenital and acquired conditions [8]. The former refers to true diverticulum, and the latter refers to muscle layer-free pseudodiverticulum. The most important features of appendiceal diverticulitis are characterized by a high perforation rate and a high incidence of concomitant malignant neoplasms, differing from acute appendicitis [7]. Therefore, at the time of preoperative diagnosis, it is important to avoid complications. Although CT is useful for the diagnosis of appendiceal diverticulitis, the preoperative diagnosis rate is low as image findings are similar to those of acute appendicitis. Early surgical treatment is recommended for appendiceal diverticulitis because conservative therapy is not effective [7].

This is the first case of MVT complicating appendiceal diverticulitis. To the best of our knowledge, no study has reported appendiceal diverticulitis complicated by MVT. Although we suspected MVT complicating appendiceal diverticulitis at the time of the visit, we also suspected other diseases, such as cholangitis, because elevated liver enzyme and hyperbilirubinemia were observed. Therefore, we initially started conservative treatment with antibiotics. Moreover, anticoagulant therapy was not performed because there was no evidence for its effectiveness in previous studies, although some studies performed it. We considered his thrombus would disappear if conservative treatment with antibiotics was successful. However, his condition deteriorated. No obvious abnormality was found in his coagulation system, and his condition improved by removing the primary disease by surgical treatment. Finally, it was inferred that appendiceal diverticulitis with high inflammation caused prothrombotic states that led to the development of MVT.

Concerning treatment for MVT complicating appendiceal diverticulitis, we suggest that surgical treatment might be effective. Of course, surgical approach is essential in cases of MVT causing significant congestion and intestinal necrosis. On the other hand, it is likely that in cases of MVT without significant congestion and intestinal necrosis, the treatment will be performed according to the treatment policy of the primary disease. Depending on the primary disease, conservative treatment might be sufficient. In fact, some studies described that the patient improved with conservative treatment when pancreatitis or inflammatory bowel disease caused MVT [9, 10]. However, the primary disease was appendiceal diverticulitis in this present case. Surgical treatment is recommended for appendiceal diverticulitis in previous studies. Ultimately, he improved after surgical treatment without anticoagulant therapy; however, many previous studies performed and recommended it. We consider that surgical treatment was sufficient for this case and might be effective for MVT complicating appendiceal diverticulitis. Further studies are needed to elucidate whether anticoagulant therapy should be administered in the perioperative period and after surgery for prophylactic treatment.

## Conclusions

This is the first case of MVT complicating appendiceal diverticulitis.

## Data Availability

Date sharing is not applicable to this article as no datasets were generated or analyzed during the current study.

## Abbreviations

CT: Computed tomography
MVT: Mesenteric venous thrombosis
WBC: White blood cell
PLT: Platelet
ALP: Alkaline phosphatase
GGT: Gamma-glutamyl transpeptidase
T-Bil: Total bilirubin
CRP: C-reactive protein
PT-INR: Prothrombin time of international normalized ration
APTT: Activated partial thromboplastin time
